# Ecology and biogeography of megafauna and macrofauna at the first known deep-sea hydrothermal vents on the ultraslow-spreading Southwest Indian Ridge

**DOI:** 10.1038/srep39158

**Published:** 2016-12-14

**Authors:** J. T. Copley, L. Marsh, A. G. Glover, V. Hühnerbach, V. E. Nye, W. D. K. Reid, C. J. Sweeting, B. D. Wigham, H. Wiklund

**Affiliations:** 1 Ocean & Earth Science, University of Southampton, Waterfront Campus, European Way, Southampton SO14 3ZH, UK; 2 Life Sciences Department, Natural History Museum, Cromwell Road, London SW7 5BD, UK; 3 National Oceanography Centre, European Way, Southampton SO14 3ZH, UK; 4 School of Biology, Newcastle University, Newcastle Upon Tyne NE1 7RU, UK; 5 Dove Marine Laboratory, School of Marine Science & Technology, Newcastle University, Cullercoats NE30 4PZ, UK

## Abstract

The Southwest Indian Ridge is the longest section of very slow to ultraslow-spreading seafloor in the global mid-ocean ridge system, but the biogeography and ecology of its hydrothermal vent fauna are previously unknown. We collected 21 macro- and megafaunal taxa during the first Remotely Operated Vehicle dives to the Longqi vent field at 37° 47′S 49° 39′E, depth 2800 m. Six species are not yet known from other vents, while six other species are known from the Central Indian Ridge, and morphological and molecular analyses show that two further polychaete species are shared with vents beyond the Indian Ocean. Multivariate analysis of vent fauna across three oceans places Longqi in an Indian Ocean province of vent biogeography. Faunal zonation with increasing distance from vents is dominated by the gastropods *Chrysomallon squamiferum* and *Gigantopelta aegis*, mussel *Bathymodiolus marisindicus*, and *Neolepas* sp. stalked barnacle. Other taxa occur at lower abundance, in some cases contrasting with abundances at other vent fields, and δ^13^C and δ^15^N isotope values of species analysed from Longqi are similar to those of shared or related species elsewhere. This study provides baseline ecological observations prior to mineral exploration activities licensed at Longqi by the United Nations.

At deep-sea hydrothermal vents, autochthonous primary production by chemosynthetic prokaryotes supports locally abundant populations of faunal species at the ocean floor. Hydrothermal vents occur as “vent fields”, each typically <10 km^2^ in extent and separated from each other by tens to hundreds of kilometres along seafloor spreading centres. Since the first investigations of hydrothermal vents in the eastern Pacific in the late 1970s, more than 250 active vent fields have been visually confirmed worldwide^1^, and more than 400 new animal species have been described from vent environments^2^ across at least eleven biogeographic provinces^3^.

The occurrence of vent fields detected along the axes of mid-ocean ridges correlates positively with seafloor spreading rate^4^,^5^. Vent fields are hundreds of kilometres apart on average along the slow-spreading Mid-Atlantic Ridge, but typically tens of kilometres apart on the fast-spreading East Pacific Rise^4^,^5^. In contrast, the longevity of hydrothermal activity at individual vent fields correlates negatively with seafloor spreading rate: geochronology of sulfides indicates activity lasting for millennia at vent fields on the Mid-Atlantic Ridge^6^, compared with decadal-scale activity at individual sites on the East Pacific Rise. These differences in the spacing and longevity of vent fields may contribute to differences in the composition and dynamics of vent fauna on different ridges^7^,^8^.

Very slow and ultraslow-spreading ridges, defined together by a full seafloor spreading rate <20 mm yr^−1^, constitute 36% of the 55 000 km global mid-ocean ridge system^9^. Faunal assemblages have only been elucidated so far at three vent fields on such ridges: one on Mohn’s Ridge in the Arctic^10^, and two associated with the Mid-Cayman Spreading Centre in the Caribbean^11^. The Mid-Cayman Spreading Centre is not geologically connected to the global mid-ocean ridge, however, and Iceland interrupts the submarine ridge system south of Mohn’s Ridge. The Southwest Indian Ridge (SWIR) forms the longest section of very slow to ultraslow-spreading seafloor in the globally contiguous mid-ocean ridge^9^, and here we report results from the first human-directed survey and sample collection at a hydrothermal vent field on this ridge.

Water column signals indicative of hydrothermal venting were detected along the Southwest Indian Ridge in 1997^12^, and the first photographs of an active vent field on the ridge were taken by an Autonomous Underwater Vehicle (AUV) in 2007^13^. The vent field, named Longqi (“Dragon’s Breath”), is located at 37° 47′S 49° 39′E and depth 2800 m. The AUV obtained images of high-temperature “black smoker” venting, sulfide deposits, and some fauna^13^, but could not collect samples. In November 2011, we therefore undertook the first dives by a Remotely Operated Vehicle (ROV) to the Longqi vent field, during Voyage 67 of the UK’s research ship *RRS James Cook*.

The aims of this study are: (1) to determine the taxonomic composition of fauna at the first known vent field on the Southwest Indian Ridge, and its biogeographic relationships with vent fauna on neighbouring seafloor spreading centres; (2) to characterise species assemblages on individual sulfide edifices with contrasting levels of hydrothermal discharge, thereby elucidating a possible successional pattern of fauna at vents on a very slow spreading ridge; and (3) to investigate the stable isotope composition of taxa at the Longqi vent field, and compare their trophic ecology with related species at other vent fields.

The Longqi vent field lies in a seabed area licensed to the Chinese Ocean Minerals Research Agency by the United Nations International Seabed Authority (ISA) in 2011 for the exploration of polymetallic sulphide mineral resources, which form at active hydrothermal vents. As ISA exploration-phase licences allow some extraction of mineral deposits to determine their composition, and testing of seabed mining technology for future exploitation-phase licensing^14^, our study also provides a baseline of ecological observations at the Longqi vent field prior to possible anthropogenic disturbances from the development of deep-sea mining^15^.

## Results

### Geomorphological features of the Longqi vent field

**Figure 1:**
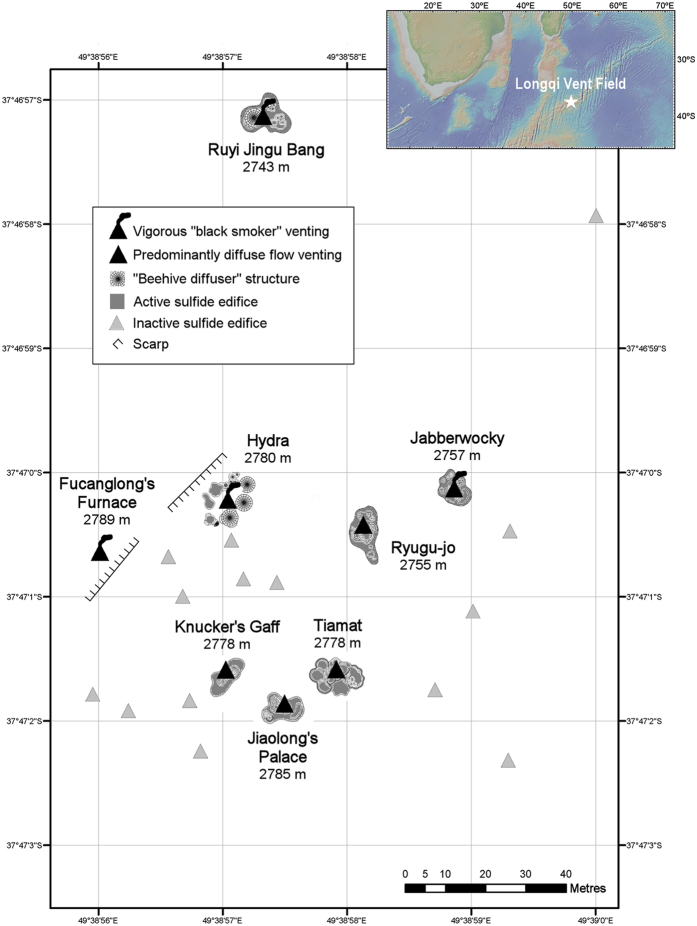
(**a**) Location map of the Longqi vent field (37° 47′S 49° 39′E) on the Southwest Indian Ridge; topography shown is from the Global Multi-Resolution Topography (GMRT) synthesis (http://www.geomapapp.org/)^60^. (**b**) Distribution of active hydrothermal vent chimneys and large inactive sulfide edifices observed during the first Remotely Operated Vehicle (ROV) dives at the Longqi vent field; depths are shown are for peaks of active chimneys measured in November 2011.

**Figure 2:**
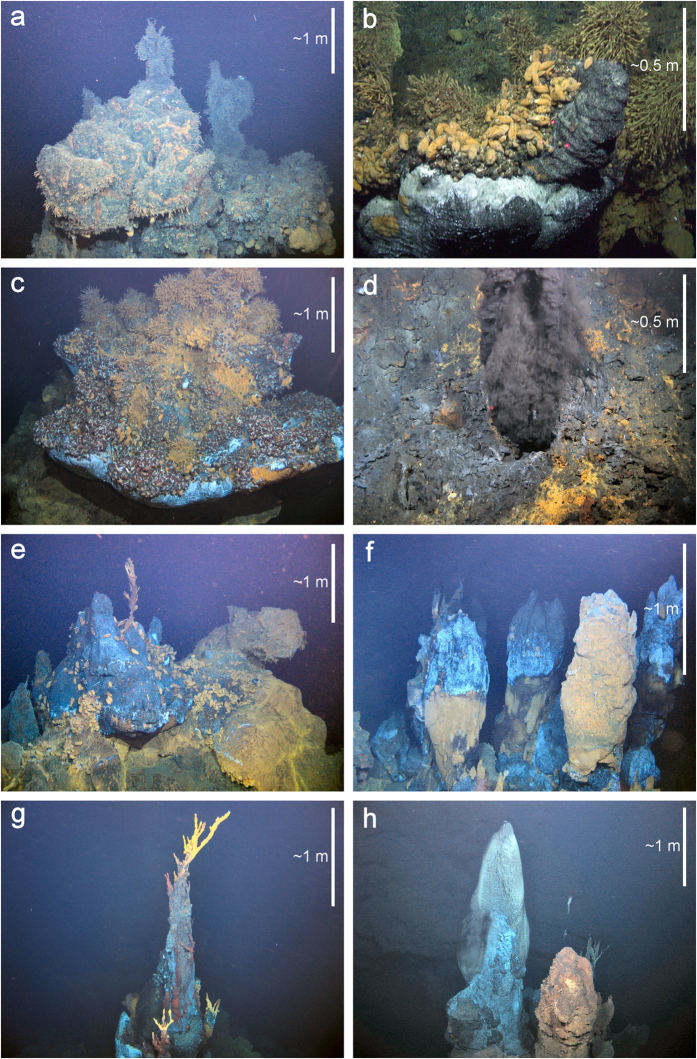
Morphology of active hydrothermal vent chimneys observed during the first Remotely Operated Vehicle (ROV) dives at the Longqi vent field, Southwest Indian Ridge, in November 2011. (**a**) “Knucker’s Gaff”; (**b**) “Jiaolong’s Palace”; (**c**) “Tiamat”; (**d**) “Fucanglong’s Furnace”; (**e**) “Ryugu-jo”; (**f**) “Hydra”; (**g**) “Jabberwocky”; (**h**) “Ruyi Jingu Bang”; locations of each edifice are shown in Fig. 1.

Our ROV survey mapped hydrothermally active and inactive sulfide edifices across an area of three hectares at the Longqi vent field (Fig. 1). Within this area, we observed eight active vent chimneys with a variety of levels of hydrothermal discharge. “Black smoker” venting, which typically requires vent fluid temperatures >300 °C^16^, was apparent at four locations: “Fucanglong’s Furnace”, “Hydra”, “Jabberwocky”, and “Ruyi Jingu Bang” (Fig. 1). These chimneys varied in the size, indicating differences in the duration of their activity. At “Fucanglong’s Furnace”, black-smoker fluids issued from a seafloor orifice with no substantial sulfide deposit (Fig. 2d), suggesting relatively recent initiation of high-temperature venting at that location. At “Hydra”, black-smoker fluids issued from a ring of six sulfide edifices ~2 m high with “beehive diffuser” structures (Fig. 2f), and “Jabberwocky” consisted of a single sulfide chimney ~6 m high topped by dendritic structures (Fig. 2g). In contrast, “Ruyi Jingu Bang” consisted of a sulfide pillar more than 20 m high, indicating more prolonged activity at that location, supporting an active “beehive diffuser”, “organ pipe” structure, and inactive sulfide spire, at its peak (Fig. 2h).

“Diffuse flow” venting of clear fluids, cooler than visible “black smoker” venting^16^, dominated hydrothermal discharge at four vent chimneys: “Ryugu-jo”, “Knucker′s Gaff”, “Jiaolong’s Palace”, and “Tiamat” (Fig. 1). These large sulfide edifices, all >15 m high, supported platforms of active and extinct “beehive diffusers” (Fig. 2a–c,e). Visible diffuse flow was less apparent at “Knucker’s Gaff”, which therefore exhibited the lowest level of venting among the active chimneys. In addition to active vent chimneys, the Longqi vent field contained at least 13 large inactive sulfide edifices, up to 15 m high, within the area surveyed by our ROV, consistent with a history of variation in the distribution of hydrothermal discharge across the vent field. Ship-towed camera systems also observed predominantly inactive sulfide deposits extending at least 1000 m north of the main vent field, as indicated by the 2007 AUV survey^13^, but with very few visible sources of vent fluids and only sparse vent fauna.

### Composition and biogeography of vent fauna on the Southwest Indian Ridge

We identified 21 macro- and megafauna taxa in samples collected from Longqi, of which seven represent previously undescribed species (Table 1). Six taxa are not known from other vent fields: *Gigantopelta aegis*^17^; *Kiwa* n. sp. “SWIR”^18^; *Peinaleopolynoe* n. sp. “Dragon”; *Ophryotrocha* n. sp. “F-038/1b”; *Phymorhynchus* n. sp. “SWIR” and *Lepetodrilus* n. sp. “SWIR” (both of which are distinct from congeners elsewhere; C Chen, pers comm). Three further taxa could not be distinguished to species level, as a consequence of a low abundance of specimens and damaged morphological condition.

**Figure 3:**
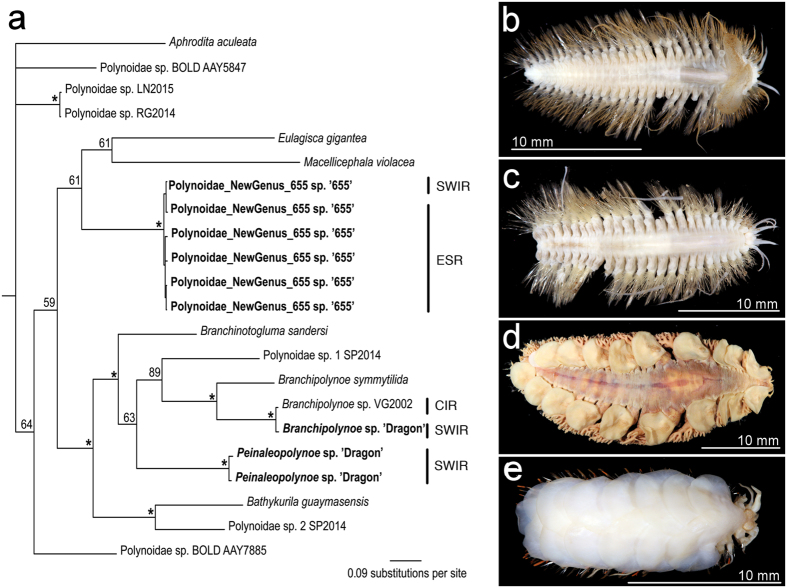
Polynoid polychaetes collected during the first Remotely Operated Vehicle (ROV) dives at Longqi vent field, Southwest Indian Ridge, in November 2011. (**a**) Bayesian phylogenetic analysis using COI marker for a limited dataset of hydrothermal vent Polynoidae (scale-worms) confirming conspecificity of a new genus and species “Polynoidae_NewGenus_655 sp. 655” at Longqi and vent fields on the East Scotia Ridge, Southern Ocean; analysis also confirms conspecificity of undescribed new species “*Branchipolynoe* sp. ‘Dragon’” at Longqi and the Karei vent field, Central Indian Ridge^21^; and the presence of an additional new species “*Peinaleopolynoe* sp. ‘Dragon’” at Longqi. (**b**,**c**) Specimens of “Polynoidae_NewGenus_655 sp. 655” collected from Longqi (**b**) and from vents on the East Scotia Ridge (**c**). (**d**) Specimen of “*Branchipolynoe* sp. ‘Dragon’” discovered at Longqi, conspecific with the Central Indian Ridge. (**e**) Specimen of “*Peinaleopolynoe* sp. ‘Dragon’” discovered at Longqi.

Six species are previously known from vent fields on the Central Indian Ridge: the “scaly-foot” gastropod *Chrysomallon squamiferum*^19^, the alvinocaridid shrimps *Rimicaris kairei*^20^ and *Mirocaris indica*^20^, the stalked barnacle *Neolepas* sp. 1^21^, and the mussel *Bathymodiolus marisindicus*^20^. *Branchipolynoe* sp. “Dragon”, a commensal scaleworm found in mussels at Longqi (Fig. 3d), appears to be conspecific on the basis of genetic similarity (Fig. 3a; 0.01 K2P and uncorrected p) with an undescribed species recorded and sequenced from the Kairei vent field on the Central Indian Ridge (“*Branchipolynoe* sp. VG-2002”)^22^. A chiridotid holothurian has also been observed at vents on the Central Indian Ridge^20^, but specimens are not yet available for comparison with the *Chiridota* sp. found at Longqi.

**Figure 4:**
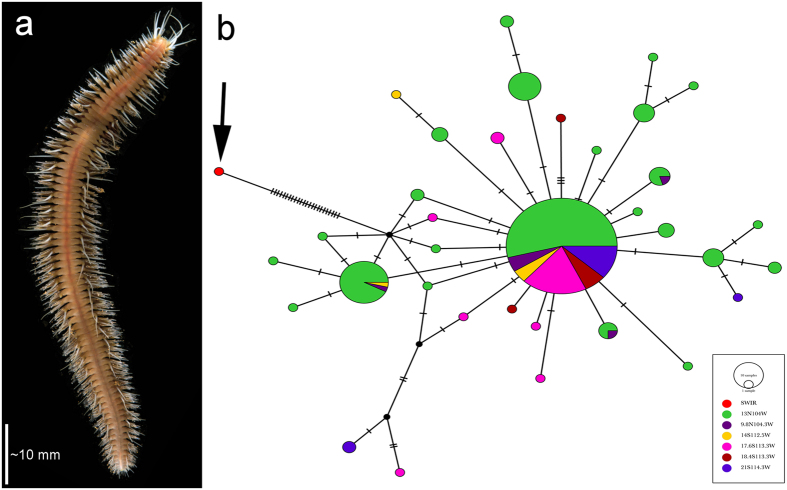
(**a**) Specimen of *Hesiolyra* cf. *bergi* collected during the first Remotely Operated Vehicle (ROV) dives at Longqi vent field, Southwest Indian Ridge, in November 2011. (**b**) Population structure analysed using TCS in PopArt using COI marker for *Hesiolyra bergi*, likely to be conspecific between Longqi and hydrothermal vent fields on the East Pacific Rise^24^; the specimen sequenced from Longqi is arrowed.

Two polychaete species found at Longqi are also present at vent fields beyond the Indian Ocean. A new genus and species of free-living scaleworm (“Polynoidae_NewGenus_655 sp. ‘655’”; Fig. 3b) is morphologically and genetically conspecific with specimens from the E2 and E9 vent fields (Fig. 3c) on the East Scotia Ridge in the Southern Ocean^3^ (Fig. 3a; 0.005–0.016 K2P and uncorrected-p between East Scotia Ridge and Longqi). A hesionid polychaete at Longqi (Fig. 4a) corresponds morphologically with *Hesiolyra bergi* from vents on the East Pacific Rise^23^. Population genetic data are available for *H. bergi* along the East Pacific Rise^24^, and including our specimens with those sequences (Fig. 4b) indicates strong population structuring between the East Pacific Rise and Southwest Indian Ridge, although these data must be considered preliminary given our single sequenced specimen. K2P and uncorrected-p distances within the East Pacific Rise populations between 13°N to 21°S are 0.01, compared with 0.07 between the Longqi and East Pacific Rise populations (Fig. 4b). We therefore use the designation *Hesiolyra* cf. *bergi* until further material is available for investigation, but note that specimens morphologically similar to *H. bergi* have also been found at vents on the Mid-Atlantic Ridge^25^, though no genetic data are available for comparison.

**Figure 5:**
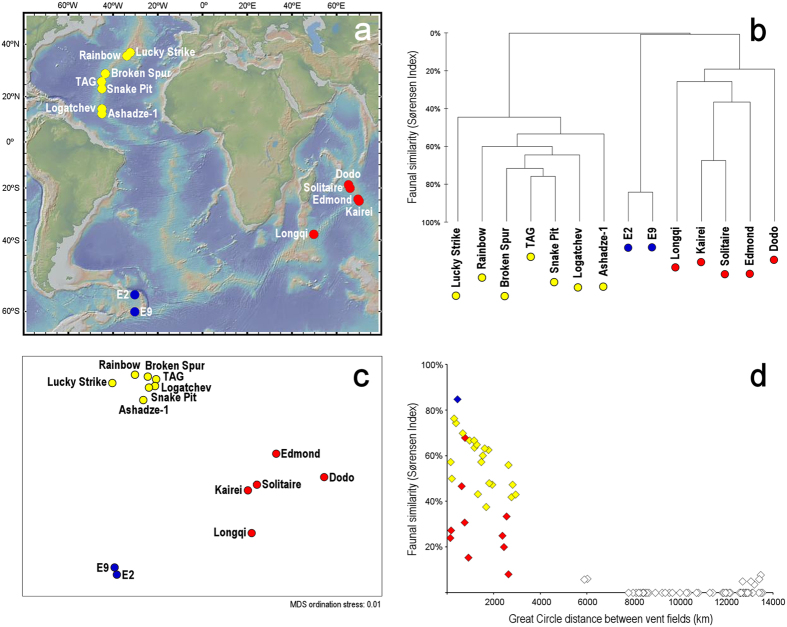
Comparison of faunal composition at Longqi vent field, Southwest Indian Ridge, with 13 well-studied vent fields on neighbouring seafloor spreading centres; red-filled circles represent vent fields in the Indian Ocean (Southwest Indian Ridge and Central Indian Ridge), yellow-filled circles represent vent fields on the Mid-Atlantic Ridge, blue-filled circles represent vent fields on the East Scotia Ridge, Southern Ocean. (**a**) Location of hydrothermal vent fields included in multivariate analysis of faunal composition; topography shown is from the Global Multi-Resolution Topography (GMRT) synthesis (http://www.geomapapp.org/)^60^. (**b**) Hierarchical agglomerative clustering using group-average linkage for presence/absence records of “chemosynthetic-environment endemic” macro- and megafaunal taxa (298 records of 139 taxa across 14 vent fields, presented as Supplementary Information). (**c**) Two-dimensional non-metric multidimensional scaling plot of Sørensen Index similarity matrix calculated from presence/absence records of “chemosynthetic-environment endemic” macro- and megafaunal taxa. (**d**) Comparison of faunal similarities between vent fields, calculated as Sørensen Index, and Great Circle distances between vent fields; yellow-filled diamonds represent pairwise comparisons among Mid-Atlantic Ridge vent fields, red-filled diamonds represent pairwise comparisons among vent fields in the Indian Ocean; blue-filled diamond represents the pairwise comparison of Southern Ocean vent fields; open diamonds represent pairwise comparisons between vent fields in different oceans, for example where a Mid-Atlantic Ridge vent field is compared with a Central Indian Ridge vent field.

Multivariate analysis of published presence/absence data for 139 macrofaunal and megafaunal taxa endemic to chemosynthetic environments from 14 well-studied vent fields in the Indian, Southern, and Atlantic Oceans (Fig. 5a; data presented in Supplementary Information) shows that the fauna at Longqi is most similar to vent fields on the Central Indian Ridge (Fig. 5b and c). This analysis also shows that vent fields within each ocean are more similar to each other in faunal composition than to those in other oceans, consistent with biogeographic provinces defined by regression tree methods^3^. Furthermore, the data reveal an overall negative correlation between faunal similarity and the spatial separation of vent fields, measured as Great Circle distances between them (Fig. 5d; r_*s*_ = −0.86, p < 0.001, n = 91 unique pairwise comparisons between 14 vent fields). Although the overall correlation is strongly influenced by low similarity values between oceans, this feature remains apparent at within-ocean scale for Atlantic vent fields (r_*s*_ = −0.61, p < 0.01, n = 21 unique pairwise comparisons between 7 vent fields).

### Faunal zonation at hydrothermal vents on the Southwest Indian Ridge

Vent chimney surfaces closest to visible high-temperature fluid sources are occupied by the alvinocaridid shrimps *Rimicaris kairei* and *Mirocaris fortunata*, *Lepetodrilus* n. sp. “SWIR” limpets, the hesionid polychaete *Hesiolyra* cf. *bergi*, the anomuran crab *Kiwa* n. sp. “SWIR”, and the “scaly-foot” gastropod *Chrysomallon squamiferum*. We only observed *Rimicaris kairei*, *Kiwa* n. sp. “SWIR”, and *Hesiolyra* cf. *bergi* in low abundance (<10 m^−2^) on vent chimneys, and *Lepetodrilus* n. sp. “SWIR” and *C. squamiferum* are therefore the first species that occur in high abundance (>100 m^−2^) with distance from vent fluid sources.

*Gigantopelta aegis* dominates the next assemblage with increasing distance from vent fluid sources, followed by aggregations of *Bathymodiolus marisindicus*, and finally *Neolepas* sp. 1. Other taxa occur at lower abundances within this zonation compared with the dominant species: for example, we only observed *Chiridota* sp. holothurians and actinostolid anemones as occasional individuals among the peripheral assemblage dominated by stalked barnacles. *Phymorhynchus* n. sp. “SWIR” gastropods also occur in this peripheral assemblage, in low abundance on sulfide edifices and in local aggregations among beds of dead mussel shells at the bases of less-active vent chimneys. *In situ* images illustrating assemblages in faunal zonation at Longqi are presented as a Supplementary Figure.

**Figure 6:**
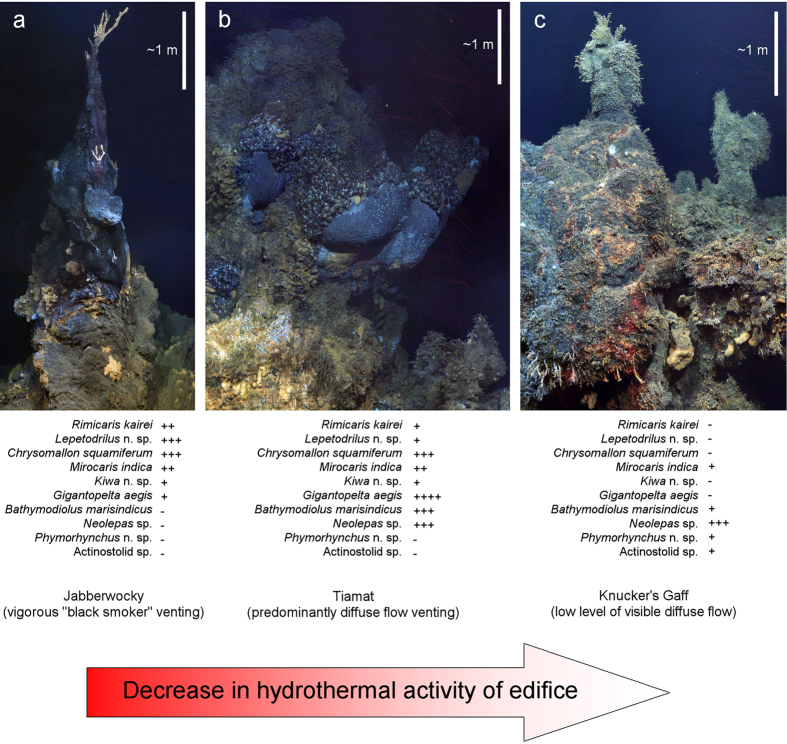
Variation in species occurrences and relative abundances on three vent chimneys with contrasting levels of hydrothermal activity at the Longqi vent field, Southwest Indian Ridge, surveyed by high-definition video mosaicking during Remotely Operated Vehicle (ROV) dives in November 2011. Relative abundances of taxa indicated as: ++++ dominant, +++ abundant, ++ common, + occasional, − not observed.

Variation in the occurrence of species on sulfide edifices with contrasting levels of hydrothermal activity, revealed by high-definition video mosaicking, suggest faunal succession as hydrothermal discharge decreases over time at individual chimneys. The “Jabberwocky” edifice represents an early stage in vent chimney evolution, with single-spire morphology and vigorous “black smoker” venting, and is occupied primarily by alvinocaridid shrimps and the scaly-foot gastropod (Fig. 6). Larger and therefore older edifices with platform morphologies and predominantly “diffuse flow” venting, such as “Tiamat”, are dominated by species from more peripheral assemblages in faunal zonation, from *Chysomallon squamiferum* to *Gigantopelta aegis*, *Bathymodiolus marisindicus*, and *Neolepas* sp. 1 (Fig. 6). *Neolepas* sp. 1 dominates the fauna at “Knucker’s Gaff”, which exhibited the lowest level of visible diffuse flow and therefore represents a waning stage of hydrothermal activity, with only occasional *Bathymodiolus marisindicus*, *Mirocaris indica*, actinostolid anemones, and *Phymorhynchus* n. sp. “SWIR” gastropods (Fig. 6).

### Stable isotope composition of taxa at Longqi vent field

**Figure 7:**
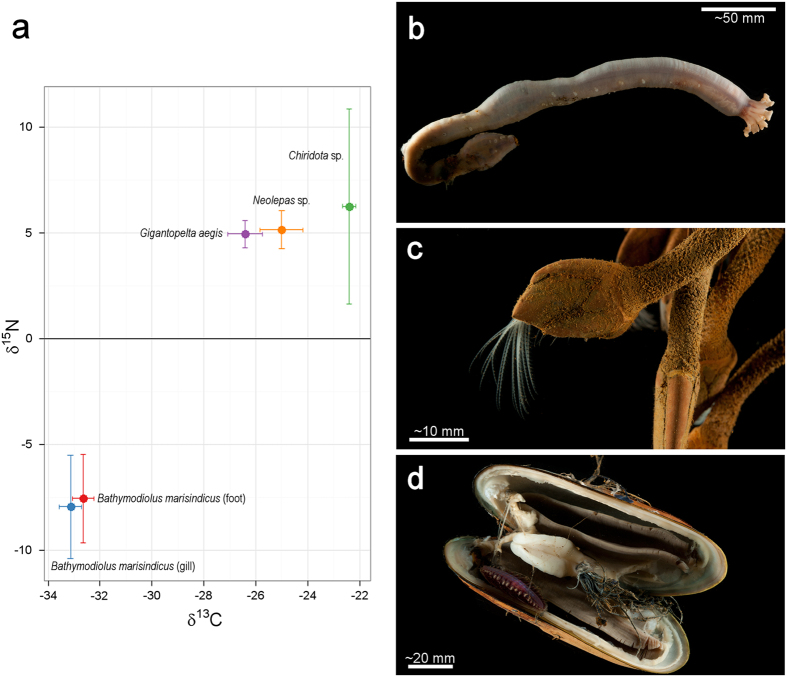
(**a**) δ13C and δ^15^N (mean and standard deviation) of taxa and tissues analysed from Longqi vent field, Southwest Indian Ridge. (**b**) Specimen of *Chiridota* sp. collected from Longqi. (**c**) Specimens of *Neolepas* sp. 1^21^ collected from Longqi. (**d**) Specimen of *Bathymodiolus marisindicus* collected from Longqi; also visible is the commensal polynoid polychaete “*Branchipolynoe* sp. ‘Dragon’” (stable isotope composition not analysed).

δ^13^C values of species analysed from Longqi ranged from −33.14‰ (±0.44) in the gills of *Bathymodiolus marisindicus* to −22.40‰ (±0.26) in the holothurian *Chiridota* sp., while *Gigantopelta aegis* (−26.42‰ ± 0.67) and *Neolepas* sp. 1 (−25.00‰ ± 0.83) were intermediate (Fig. 7). Foot and gill tissue δ^13^C from *B. marisindicus* were similar (−32.64‰ ± 0.41 and −33.14‰ ± 0.44 respectively). Paired δ^15^N of taxa analysed from Longqi ranged between −7.95‰ (±2.45) and 6.27‰ (±4.61), with mussel gills having the lowest δ^15^N and *Chiridota* sp. the highest. *Gigantopelta aegis* and *Neolepas* sp. 1 were similar and intermediate in δ^15^N (Fig. 7), with values of 4.96‰ (±0.64) and 5.16‰ (±0.91) respectively.

## Discussion

Longqi is ecologically distinct among known hydrothermal vent fields, hosting species not yet recorded from other locations, and known species in abundances that contrast with populations elsewhere. The species richness of 21 mega- and macrofaunal taxa in our samples is within the range of values for well-studied vent fields on neighbouring seafloor spreading centres (4 to 35 taxa at vent fields on the Central Indian Ridge^2^,^20^,^22^,^26^; 17 to 43 taxa at Mid-Atlantic Ridge vent fields^2^,^25^,^27^,^28^,^29^,^30^; 12 to 14 taxa at vents on the East Scotia Ridge^3^,^31^,^32^,^33^; see Supplementary Information for full details), providing confidence of adequate sampling at Longqi for comparative analysis in this study.

The majority of known mega- and macrofaunal species found at Longqi are previously recorded from the Central Indian Ridge, with which this Southwest Indian Ridge vent field therefore has closest affinity in species composition. COI gene sequence data reveal significant differentiation, however, between Southwest Indian Ridge and Central Indian Ridge populations of the scaly-foot gastropod *Chrysomallon squamiferum*^34^, consistent with low connectivity across the ~2300 km between those sites via the lecithotrophic larvae inferred for this species^19^. The extent of contemporary connectivity has yet to be determined between Southwest Indian Ridge and Central Indian Ridge populations of species with planktotrophic larval development such as *Rimicaris kairei*, whose congener *R. exoculata* exhibits panmixia in microsatellite markers over a distance of ~7100 km among vent fields in the Atlantic^35^.

Several species in our samples from Longqi exhibit an affinity at higher taxonomic level with seafloor spreading centres beyond the Indian Ocean. *Kiwa* n. sp. “SWIR” is morphologically most similar among the Kiwaidae to *K. tyleri*^32^ from the East Scotia Ridge, with a molecular phylogeny based on nine gene sequences indicating divergence at 2.6 to 0.6 (median 1.5) Ma^18^. Similarly, *Gigantopelta aegis* is closely related to *G. chessoia* from the East Scotia Ridge, with 4.43% COI divergence and molecular clock calibrations suggesting a common ancestor around 1.85 to 1.54 Ma^17^. Among eolepadid barnacles, a split between *Neolepas* sp. 1 and *Vulcanolepas scotiaensis* of the East Scotia Ridge is also indicated at 3.8 to 0.4 (median 1.7) Ma^21^. Changes in the latitudinal range of the Antarctic Circumpolar Current, such as those inferred between 1.2 Ma and 650 ka, may have increased hydrographic isolation of the Southwest Indian Ridge from the East Scotia Ridge^18^, possibly contributing to the allopatric speciation of these taxa. A chiridotid holothurian has been reported at vents on the Central Indian Ridge^20^, and *Chiridota hydrothermica* is known at vents in the back-arc basins of the western Pacific and on the southern East Pacific Rise in similar distribution and abundance to the species at Longqi^36^, but further comparison is required to confirm the affinity of the species on the SW Indian Ridge.

The discovery of a polynoid species at Longqi shared with vent fields ~6000 km away on the East Scotia Ridge, however, and *Hesiolyra* cf. *bergi* potentially shared with the East Pacific Rise, is consistent with the most widely-distributed species at hydrothermal vents being polychaetes. The amphinomid species *Archinome tethyana* and *A. jasoni*, for example, have been found at vents on the Mid-Atlantic Ridge and the Central Indian Ridge^37^. These trans-oceanic polychaete species are therefore responsible for the “non-zero” faunal similarity values between some vent fields in different biogeographic provinces (Fig. 6d). The potential trans-oceanic distribution of *H. bergi* may be extended further if future studies confirm that the hesionid resembling *H. bergi* on the Mid-Atlantic Ridge^25^ is conspecific with populations on the Southwest Indian Ridge and East Pacific Rise. Similarly, we identified a spionid specimen from Longqi as *Prionospio* cf. *unilamellata* (Table 1) on the basis of morphology, and *P. unilamellata* is known from Mid-Atlantic vents^25^, but paucity of material prevented more detailed morphological investigation or molecular analysis.

A negative correlation between faunal similarity and along-ridge-axis distance between vent fields has previously been noted at genus level^38^, and here we show an overall negative correlation between species-level faunal similarity and Great Circle distances between vent fields across three ocean regions (Fig. 5d). This relationship may be weaker, however, where neighbouring vent fields vary in levels of hydrothermal activity as a result of their ephemeral nature. The “Dodo” vent field on the intermediate-spreading Central Indian Ridge, for example, is waning in activity compared with the nearby “Solitaire” vent field^26^, and these vent fields consequently differ markedly in faunal composition (Sørensen’s Index 24%) despite being only 145 km apart (Fig. 5a). Such variation may be less likely on slower-spreading ridges, however, where individual vent fields exhibit greater longevity of hydrothermal activity^6^, and this may contribute to the negative correlation remaining apparent among vents on the Mid-Atlantic Ridge (Fig. 5d).

The extensive inactive sulfide deposits at Longqi are consistent with a prolonged history of hydrothermal activity at the vent field, as expected on a very slow spreading ridge. Our comparison of species on chimneys with contrasting levels of hydrothermal activity suggests that when activity wanes for an individual chimney, its fauna will follow a temporal succession that matches the spatial zonation around the vents. The low abundance of *Rimicaris kairei* on active vent chimneys at Longqi contrasts with the high-abundance aggregations of this species in the same environment at vents on the Central Indian Ridge^20^,^22^,^26^, and the low abundance of *Kiwa* n. sp. “SWIR” close to vent fluid sources also contrasts with the aggregations of closely-related *K. tyleri* in the same location in zonation at vents on the East Scotia Ridge^39^. We did not observe the large provannid gastropod *Alviniconcha hessleri*, which occurs in high abundance at several vent fields on the Central Indian Ridge^20^,^22^,^26^. More peripheral taxa in the faunal zonation at Longqi, however, occur in comparable abundances to populations of shared or related species elsewhere, such as the aggregations of *Gigantopelta aegis* resembling those of closely-related *G. chessoia* at vents on the East Scotia Ridge^39^, and *Neolepas* sp. 1 occurring in high abundance as found at vents on the Central Indian Ridge^20^,^21^,^22^,^26^.

Despite differences in overall faunal composition compared with vent fields on other ridges, carbon and nitrogen stable isotope composition of species analysed from Longqi are generally similar to those of shared or related species elsewhere, suggesting similar trophic roles. *Bathymodiolus* gill and foot δ^13^C values are at the upper range of values expected from carbon fixed by the Calvin Benson Bassham cycle, and may also contain contributions of organic carbon produced by methane-oxidisers^40^, consistent with dual endosymbiosis known in bathymodiolin mussels elsewhere^41^,^42^,^43^,^44^,^45^,^46^. δ^13^C values of *Gigantopelta aegis* are similar to those of *G. chessoia* on the East Scotia Ridge^47^ (reported as Peltospiroidea sp.), and δ^13^C values of *Neolepas* sp. 1 are similar to *Vulcanolepas scotianesis* on that ridge^47^ (reported as *Vulcanolepas* sp.). The values for *Neolepas* sp. 1 are lower than conspecific values at the Kairei vent field on the Central Indian Ridge^45^ (~−16‰), however, indicating possible site-specific differences in composition or δ^13^C values of microbial food sources. The highest δ^13^C observed among species analysed from Longqi were in *Chiridota* sp., similar to the values found in a chiridotid holothurian at the Solwara-1 vent field in the western Pacific^48^ (δ^13^C = ~−24‰).

*Bathymodiolus* was the only taxon analysed from Longqi with negative δ^15^N values, which are mid-range among those reported for bathymodiolin mussels at hydrothermal vents (~−17‰ to ~6‰)^49^,^50^. Positive δ^15^N values of *Gigantopelta aegis*, *Neolepas* sp. 1, and *Chiridota* sp. are within ~1.3‰ of each other, indicating a comparable inorganic nitrogen source. *G. aegis* δ^15^N is similar to that of other large peltospirid gastropods^46^,^47^,^51^, and δ^15^N of *Neolepas* sp. 1 at Longqi is within the range for stalked barnacles at other hydrothermal vents (~5‰ to ~11‰)^46^,^47^,^48^.

As this is the first ecological investigation of hydrothermal vents on the Southwest Indian Ridge, further exploration is needed to determine whether the faunal assemblage at Longqi is typical of vent fields on this very slow to ultraslow-spreading ridge. Until such information is available, the Longqi vent field appears to meet several criteria that may define an “Ecologically or Biologically Sensitive Area” under the UN Convention on Biological Diversity (CBD), for example an area that “contains unique, rare, or endemic species, populations or communities”^52^. Assessing the impacts of mineral exploration activities already licensed at Longqi by the UN International Seabed Authority (ISA)^14^ should therefore include investigation of other vent fields detected on the Southwest Indian Ridge and the relationships of their fauna with populations at Longqi.

## Methods

### Deep-sea sampling and surveying

The *Kiel6000* ROV undertook three dives to the Longqi vent field during 27 to 30 November 2011, spending a total of 22 hours at the seafloor^53^. A towed camera system (*SHRIMP* – Seabed High Resolution Imaging Platform) and manoeuvrable TV grab system (*HyBIS* – Hydraulic Benthic Interactive Sampler) were also used to examine the area of predominately inactive hydrothermal deposits extending ~1 km to the north of the active vent field. A shipboard ultrashort baseline (USBL) acoustic system provided vehicle navigation for mapping the locations of seabed features during dives.

Faunal specimens were collected using a suction sampler and scoops deployed by the ROV’s manipulators at five separate locations^53^, chosen to provide representative samples of assemblages seen in faunal zonation. Each sample from a different location was segregated in an individual collection container aboard the vehicle. After each dive, samples were sieved at 250 μm, immediately transferred to a 4 °C constant-temperature laboratory aboard ship, and sorted into morphospecies. Specimens for morphological studies were fixed in seawater-buffered 4% formaldehyde, while specimens for molecular analyses were preserved in 100% ethanol, and specimens for stable isotope analysis were frozen at −80 °C.

Three sulfide edifices with different levels of visible hydrothermal activity (“Jabberwocky”; “Tiamat”; and “Knucker’s Gaff”) were targeted for high-definition video mosaicking of their vertical faces. Closed-loop control using Doppler-velocity log data enabled the ROV to manoeuvre in a precise vertical plane facing each vent chimney^54^, recording digital video in uncompressed ProRes 422 format from a forward-facing camera with parallel lasers providing a 0.1 m scale. High-definition video frames were extracted from this footage and processed to produce composite images of the sulfide edifices^54^ (Fig. 7). Occurrences of macrofaunal species were noted in these composite images for each chimney, and their relative abundances estimated for each chimney using Dominant-Abundant-Common-Occasional categories.

### Molecular phylogenetic and population genetic analyses

DNA extraction and phylogenetic analyses are described elsewhere for *Kiwa* n. sp. “SWIR”^18^, *Chrysomallon squamiferum*^19^, *Gigantopelta aegis*^17^, and *Neolepas* sp. 1^21^ from Longqi. For the polynoid polychaetes and *Hesiolyra* cf. *bergi* reported here, DNA was extracted using Qiagen DNeasy Blood and Tissue Kit following the protocol from the manufacturer. Approximately 660 bp of the mitochondrial gene COI were amplified using the primers LCO1490 5′-GGTCAACAAATCATAAAGATATTGG-3′ and HCO2198 5′-TAAACTTCAGGGTGACCAAAAAATCA-3′^55^. PCR mixtures contained 1 μl of each primer (10μM), 2 μl of DNA template, and 21 μl of Red Taq DNA Polymerase 1.1X MasterMix (VWR). The PCR profile was 94 °C/300s, (94 °C/60 s, 55 °C/60 s, 72 °C/120 s) × 35 cycles, 72 °C/300 s. PCR purification was done using a Millipore Multiscreen 96-well PCR Purification System, and sequencing was performed on an ABI 3730XL DNA Analyser (Applied Biosystems) at the Natural History Museum Sequencing Facility, using the primers mentioned above.

Overlapping sequence fragments were concatenated into consensus sequences using Geneious v.6.1.7^56^, and aligned using the MUSCLE plug-in with default settings. Bayesian molecular phylogenetic analyses were conducted using MrBayes 3.1.2^57^ for the polynoid polychaetes, and the haplotype network for *Hesiolyra cf. bergii* was constructed using TCS in PopART (http://popart.otago.ac.nz). The COI dataset of 670 bp was run three times for 10 million generations, with 2.5 million generations discarded as burn-in. Average genetic distances within and amongst inferred clades were calculated using uncorrected p-distance and Kimura two parameter (K2P) models implemented in Mesquite v.3.04 (http://mesquiteproject.org). DNA sequences have been deposited in NCBI GenBank with the following accession numbers: KY211993 (*Branchipolynoe* n. sp. “Dragon”), KY211994 (*Hesiolyra* cf. *bergi*), KY211995 (*Ophryotrocha* n. sp. “F-038/1b”), KY211996 (*Peinaleopolynoe* n. sp. “Dragon”), KY211997 (Polynoidae n. gen. n. sp. “655”).

### Multivariate analysis of faunal similarity with vent fields on neighbouring seafloor spreading centres

To examine the biogeographic context of vent fauna at Longqi, the species list for the site (Table 1) was compared with species lists compiled from published literature for 13 well-studied vent fields on neighbouring seafloor spreading centres: the Central Indian Ridge (Kairei, Edmond, Solitaire, and Dodo fields^2^,^20^,^22^,^26^); the East Scotia Ridge (E2 and E9 fields^3^,^17^,^31^,^32^,^33^); and Mid-Atlantic Ridge (Lucky Strike, Rainbow, Broken Spur, TAG, Snake Pit, Ashadze-1, and Logatchev fields^2^,^25^,^27^,^28^,^29^,^30^). Meiofaunal taxa were excluded, as meiofaunal species have not always been sampled or characterised in samples from vents, and therefore their true absence cannot be inferred reliably from literature for each vent field. “Non-vent” taxa (defined as species originally described from non-chemosynthetic environments) were also excluded for the same reason, as such “normal” deep-sea taxa on the periphery of vent fields are not consistently included in species lists published for different sites. The omission of these variably recorded groups therefore helps to ensure equivalent datasets from each vent field for comparative analyses, by only considering presence/absence of “chemosynthetic-environment endemic” macro- and megafaunal taxa.

Identities were defined to species level where possible, and indeterminate species of the same genus at different sites were conservatively assigned to separate taxonomic units to avoid potential false conflation of faunal similarity. In total, the resulting database of vent fauna (presented as Supplementary Information) contains 298 records of 139 taxa across 14 vent fields. A similarity matrix between vent fields was calculated from taxon presence/absence records using Sørensen’s Index^58^. Hierarchical agglomerative clustering using group-average linkage, and non-metric multidimensional scaling, were applied to the similarity matrix using PRIMER version 6 (PRIMER-E, Plymouth UK)^59^ to produce a dendrogram and two-dimensional ordination representing similarity relationships (Fig. 5b,c). To examine possible correlations between geographic separation and faunal similarity (Fig. 5d), “Great Circle” distances between vent fields were calculated from their latitude and longitude coordinates.

### Stable isotope analyses

Specimens collected for stable isotope analyses were defrosted ashore, dissected to remove tissue for analysis, rinsed with distilled water and refrozen at −80 °C. Tissue samples were freeze-dried and ground to a fine homogenous powder using a pestle and mortar. Approximately 1 mg of powder was weighed into a tin capsule for dual carbon and nitrogen stable isotope analysis using an elemental analyser coupled to a Europa Scientific 20–20 isotope ratio mass spectrometer (Iso-Analytical, Crewe, United Kingdom). The laboratory standards for calibration and drift correction were powdered bovine liver (δ^13^C) and AIR (δ^15^N). Internal standards of beet sugar, cane sugar, and ammonium sulfate were used for quality control. All internal standards are traceable to the following international standards: NBS-1577B, IAEA-CH-6 (sucrose), and IAEA-N-1 (ammonium sulfate). Stable isotope ratios were expressed in delta (δ) notation as parts per thousand/per mil (‰). An external standard of freeze-dried and ground fish muscle (*Antimora rostrata*) was also analysed (n = 3; δ^13^C: −18.74 ± s.d. 0.03; δ^15^N: 13.33 ± 0.004 s.d.).

## Additional Information

**How to cite this article**: Copley, J. T. *et al*. Ecology and biogeography of megafauna and macrofauna at the first known deep-sea hydrothermal vents on the ultraslow-spreading Southwest Indian Ridge. *Sci. Rep.* **6**, 39158; doi: 10.1038/srep39158 (2016).

**Publisher’s note:** Springer Nature remains neutral with regard to jurisdictional claims in published maps and institutional affiliations.

## Supplementary Material

Supplementary Information

## Figures and Tables

**Table 1:** Taxa identified in faunal samples collected during the first Remotely Operated Vehicle (ROV) dives at the Longqi vent field, Southwest Indian Ridge, in November 2011.

Phylum	Class	Taxon	Presence on other ridges
Cnidaria	Anthozoa	Actinostolidae sp.	
Annelida	Polychaeta	Polynoidae n. gen. n. sp. “655”	ESR (Fig. 4)
		*Branchipolynoe* n. sp. “Dragon”	CIR (Fig. 4)
		*Peinaleopolynoe* n. sp. “Dragon”	
		*Hesiolyra* cf. *bergi*	EPR (Fig. 5)^23^
		Hesionidae sp. indet.	
		*Ophryotrocha* n. sp. “F-038/1b”	
		*Prionospio* cf. *unilamellata*	
		Ampharetidae sp. indet.	
Mollusca	Bivalvia	*Bathymodiolus marisindicus*	CIR^20^
	Gastropoda	*Chrysomallon squamiferum*	CIR^19^
		*Gigantopelta aegis*^17^	
		*Phymorhynchus* n. sp. “SWIR” (distinct from CIR species; C Chen pers comm)	
		*Lepetodrilus* n. sp. “SWIR” (distinct from CIR species; C Chen pers comm)	
Arthropoda	Maxillopoda	*Neolepas* sp. 1	CIR^21^
	Malacostraca	*Rimicaris kairei*	CIR^20^
		*Mirocaris indica*	CIR^20^
		*Chorocaris* sp.	
		*Kiwa* n. sp. “SWIR”^17^	
		*Munidopsis* sp.	
Echinodermata	Holothuroidea	*Chiridota* sp.	

Species presence on other ridges indicated as: ESR = East Scotia Ridge; CIR = Central Indian Ridge; EPR = East Pacific Rise.
