# Supplementary material for: Ecology and biogeography of megafauna and macrofauna at the first known deep-sea hydrothermal vents on the ultraslow-spreading Southwest Indian Ridge
Source: Sci Rep. 2016 Dec 14;6:39158. doi: 10.1038/srep39158 (PMC5155287; doi:10.1038/srep39158)
Supplement: Supplementary Information [file srep39158-s1.pdf]

SUPPLEMENTARY INFORMATION

Ecology and biogeography of megafauna and macrofauna at the first known deep-sea hydrothermal vents on the ultraslow-spreading Southwest Indian Ridge

Copley JT[1,*], Marsh L[1], Glover AG[2], Hühnerbach V[3], Nye VE[1], Reid WDK[4], Sweeting CJ[5], Wigham BD[5], Wiklund H[2]

[1]Ocean & Earth Science, University of Southampton, Waterfront Campus, European Way, Southampton SO14 3ZH, UK

[2]Life Sciences Department, Natural History Museum, Cromwell Road, London SW7 5BD, UK

[3]formerly at National Oceanography Centre, European Way, Southampton SO14 3ZH, UK

[4]School of Biology, Newcastle University, Newcastle Upon Tyne NE1 7RU, UK

[5]Dove Marine Laboratory, School of Marine Science & Technology, Newcastle University, Cullercoats NE30 4PZ, UK

*email jtc@southampton.ac.uk (corresponding author)

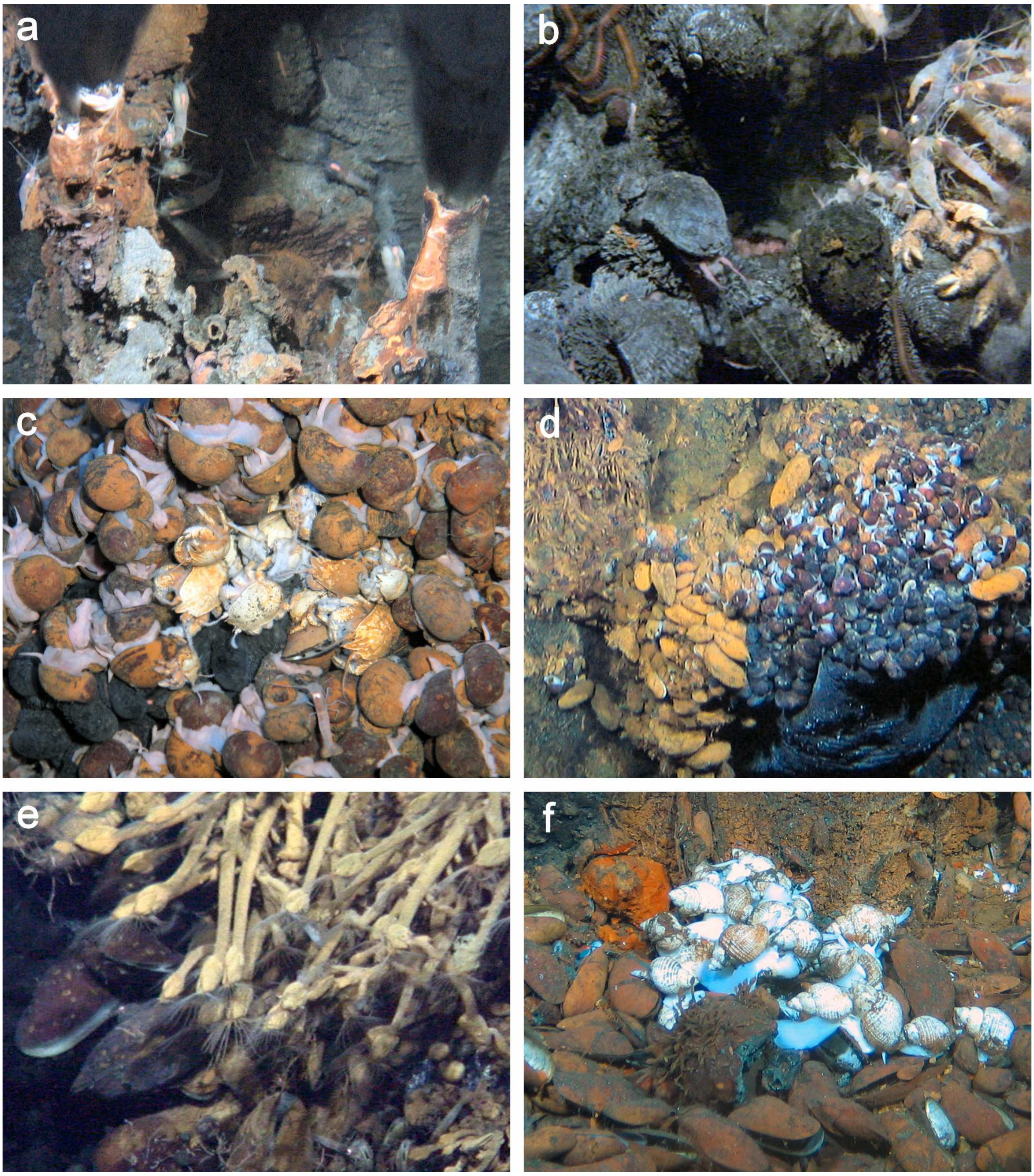

SUPPLEMENTARY FIGURE: Images of faunal assemblages observed at Longqi vent field, Southwest Indian Ridge, during the first remotely operated vehicle (ROV) dives in November 2011: (a) active “black smoker” chimneys occupied by *Rimicaris kairei*; (b) assemblage of *Chrysomallon squamiferum*, *Hesiolyra* cf. *bergi*, *Kiwa* n. sp. “SWIR”, *Mirocaris fortunata* in close proximity to vent fluid source; (c) abundant *Chrysomallon squamiferum* and *Gigantopelta aegis*, with *Kiwa* n. sp. “SWIR”, *Bathymodiolus marisindicus*, and *Mirocaris fortunata* on platform of “Tiamat” vent chimney (d) zonation of *Chysomallon squamiferum*, *Gigantopelta aegis*, *Bathymodiolus marisindicus*, and *Neolepas* sp. 1 with distance from actively venting beehive diffuser; (e) transition between aggregations of *Bathymodiolus marisindicus* and *Neolepas* sp. 1; (f) aggregation of *Phymorhynchus* n. sp. “SWIR” associated with dead mussel shells at base of active vent chimney.

SUPPLEMENTARY DATA: presence/absence data for "chemosynthetic-environment endemic" macro- and megafaunal taxa at Longqi vent field on the Southwest Indian Ridge and 13 well-studied hydrothermal vent fields on neighbouring seafloor spreading centres, compiled from the literature (Central Indian Ridge: Kairei, Edmond, Solitaire, and Dodo fields[2,20,22,26]; East Scotia Ridge, Southern Ocean: E2 and E9 fields[3,17,31,32,33]; Mid-Atlantic Ridge: Lucky Strike, Rainbow, Broken Spur, TAG, Snake Pit, Ashadze-1, and Logatchev fields[2,25,27,28,29,30]).

					SW Indian Ridge	*Central Indian Ridge*				*Mid-Atlantic Ridge*							*East Scotia Ridge*	
**Phylum**	**Class**	**other**	**Family**	**Species**	**Longqi**	**Kairei**	**Edmond**	**Solitaire**	**Dodo**	**Lucky Strike**	**Rainbow**	**Broken Spur**	**TAG**	**Snake Pit**	**Logatchev**	**Ashadze-1**	**E2**	**E9**
Cnidaria	Anthozoa		Actinostolidae	***Maractis rimicarivora***	0	0	0	0	0	1	1	1	1	1	0	1	0	0
Cnidaria	Anthozoa		Actinostolidae	***Marianactis* sp.**	0	1	0	1	1	0	0	0	0	0	0	0	0	0
Cnidaria	Anthozoa		Actinostolidae	**Actinostolid sp. SWIR**	1	0	0	0	0	0	0	0	0	0	0	0	0	0
Cnidaria	Anthozoa		Actinostolidae	**Actinostolid sp. ESR1**	0	0	0	0	0	0	0	0	0	0	0	0	0	1
Cnidaria	Anthozoa		Actinostolidae	**Actinostolid sp. ESR2**	0	0	0	0	0	0	0	0	0	0	0	0	1	1
Cnidaria	Anthozoa		Actinostolidae	**Actinostolid sp. ESR3**	0	0	0	0	0	0	0	0	0	0	0	0	1	1
Cnidaria	Anthozoa		Actinostolidae	**Actinostolid sp. ESR4**	0	0	0	0	0	0	0	0	0	0	0	0	1	0
Annelida	Polychaeta		Alvinellidae	**Alvinellid sp.**	0	0	1	1	0	0	0	0	0	0	0	0	0	0
Annelida	Polychaeta		Ampharetidae	***Amathys lutzi***	0	0	0	0	0	1	1	1	0	1	1	1	0	0
Annelida	Polychaeta		Ampharetidae	***Glyphanostomum* sp.**	0	0	0	0	0	0	0	0	0	0	0	1	0	0
Annelida	Polychaeta		Ampharetidae	**Ampharetid sp. indet.**	1	0	0	0	0	0	0	0	0	0	0	0	0	0
Annelida	Polychaeta		Amphinomidae	***Archinome jasoni***	0	1	0	1	0	0	0	0	0	0	1	0	0	0
Annelida	Polychaeta		Amphinomidae	***Archinome tethyana***	0	0	1	0	0	0	0	0	1	1	0	1	0	0
Annelida	Polychaeta		Amphinomidae	***Archinome* sp. indet. Rainbow**	0	0	0	0	0	0	1	0	0	0	0	0	0	0
Annelida	Polychaeta		Amphinomidae	***Archinome* sp. indet. Broken Spur**	0	0	0	0	0	0	0	1	0	0	0	0	0	0
Annelida	Polychaeta		Amphinomidae	***Archinome* sp. indet. Edmond**	0	0	1	0	0	0	0	0	0	0	0	0	0	0
Annelida	Polychaeta		Amphinomidae	***Amphisamytha* sp.**	0	1	0	0	0	0	0	0	0	0	0	0	0	0
Annelida	Polychaeta		Capitellidae	**Capitellid sp. CIR**	0	1	0	0	0	0	0	0	0	0	0	0	0	0
Annelida	Polychaeta		Capitellidae	**Capitellid sp. MAR1**	0	0	0	0	0	1	0	0	0	0	0	0	0	0
Annelida	Polychaeta		Capitellidae	**Capitellid sp. MAR2**	0	0	0	0	0	0	0	0	0	0	0	1	0	0
Annelida	Polychaeta		Chaetopteridae	***Spirochaetopterus* sp.**	0	0	0	0	0	0	1	0	1	0	0	0	0	0
Annelida	Polychaeta		Chaetopteridae	***Phyllochaetopterus* sp.**	0	0	0	0	0	0	0	0	0	0	0	1	0	0
Annelida	Polychaeta		Cirratulidae	**Cirratulid sp. MAR**	0	0	0	0	0	0	0	0	0	0	0	1	0	0
Annelida	Polychaeta		Dorvilleidae	**Dorvilleid sp. MAR**	0	0	0	0	0	1	0	0	0	0	0	0	0	0
Annelida	Polychaeta		Dorvilleidae	**Dorvilleid sp. CIR**	0	1	0	0	0	0	0	0	0	0	0	0	0	0
Annelida	Polychaeta		Dorvilleidae	***Ophryotrocha* n. sp. "F-038/1b"**	1	0	0	0	0	0	0	0	0	0	0	0	0	0
Annelida	Polychaeta		Hesionidae	**Hesionid sp. MAR**	0	0	0	0	0	0	0	0	0	0	0	1	0	0
Annelida	Polychaeta		Hesionidae	**cf. *Hesiolyra* sp.**	0	0	0	0	0	1	0	0	0	0	0	0	0	0
Annelida	Polychaeta		Hesionidae	**Hesionid sp. indet.**	1	0	0	0	0	0	0	0	0	0	0	0	0	0
Annelida	Polychaeta		Hesionidae	***Hesiolyra* cf. *bergi***	1	0	0	0	0	0	0	0	0	0	0	0	0	0
Annelida	Polychaeta		Hesionidae	**Hesionid sp. CIR1**	0	1	0	0	0	0	0	0	0	0	0	0	0	0
Annelida	Polychaeta		Hesionidae	**Hesionid sp. CIR2**	0	1	0	0	0	0	0	0	0	0	0	0	0	0
Annelida	Polychaeta		Iphionidae	***Thermiphione* sp.**	0	0	0	0	0	0	0	0	0	0	0	1	0	0
Annelida	Polychaeta		Polynoidae	***Branchinotogluma mesatlantica***	0	0	0	0	0	1	0	0	0	0	0	0	0	0
Annelida	Polychaeta		Polynoidae	***Branchinotogluma* sp. CIR**	0	1	0	1	0	0	0	0	0	0	0	0	0	0
Annelida	Polychaeta		Polynoidae	***Branchipolynoe* n. sp. "Dragon"**	1	1	1	1	0	0	0	0	0	0	0	0	0	0
Annelida	Polychaeta		Polynoidae	***Branchipolynoe* sp. CIR**	0	1	0	0	0	0	0	0	0	0	0	0	0	0
Annelida	Polychaeta		Polynoidae	***Branchipolynoe seepensis***	0	0	0	0	0	1	1	1	0	1	1	0	0	0
Annelida	Polychaeta		Polynoidae	***Peinaleopolynoe n.* sp. "Dragon"**	1	0	0	0	0	0	0	0	0	0	0	0	0	0
Annelida	Polychaeta		Polynoidae	***Lepidonotopodium jouinae***	0	0	0	0	0	1	0	0	0	0	0	0	0	0
Annelida	Polychaeta		Polynoidae	***Levensteiniella iris***	0	0	0	0	0	1	1	0	0	0	0	1	0	0
Annelida	Polychaeta		Polynoidae	**cf. *Levensteiniella* sp.**	0	1	0	0	0	0	0	0	0	0	0	0	0	0
Annelida	Polychaeta		Polynoidae	**cf. *Harmothoe***	0	1	0	0	0	0	0	0	0	0	0	0	0	0
Annelida	Polychaeta		Polynoidae	**Polynoidae gen. nov. sp. nov. "655"**	1	0	0	0	0	0	0	0	0	0	0	0	1	1
Annelida	Polychaeta		Spionidae	***Laonice athecata***	0	0	0	0	0	1	0	0	0	0	1	0	0	0
Annelida	Polychaeta		Spionidae	***Prionospio unilamellata***	0	0	0	0	0	1	1	0	0	1	0	0	0	0
Annelida	Polychaeta		Spionidae	***Prionospio* cf. *unilamellata***	1	0	0	0	0	0	0	0	0	0	0	0	0	0
Annelida	Polychaeta		Spionidae	***Prionospio* sp. CIR**	0	1	0	0	0	0	0	0	0	0	0	0	0	0
Annelida	Polychaeta		Spionidae	***Prionospio* sp. MAR**	0	0	0	0	0	0	0	0	0	0	0	1	0	0
Mollusca	Solenogastres		Simrothiellidae	**cf. *Helicoradomenia* sp.**	0	1	0	0	0	0	0	0	0	0	0	0	0	0
Mollusca	Gastropoda		Dendronotidae	***Dendronotus comteti***	0	0	0	0	0	1	0	0	0	0	0	0	0	0
Mollusca	Gastropoda		Elachisinidae	***Laeviphitus desbruyeresi***	0	0	0	0	0	1	1	0	0	0	0	0	0	0
Mollusca	Gastropoda		Lepetodrilidae	***Lepetodrilus atlanticus***	0	0	0	0	0	1	0	1	1	1	0	1	0	0
Mollusca	Gastropoda		Lepetodrilidae	***Lepetodrilus concentricus***	0	0	0	0	0	0	0	0	0	0	0	0	1	1
Mollusca	Gastropoda		Lepetodrilidae	***Lepetodrilus* sp. A**	1	0	0	0	0	0	0	0	0	0	0	0	0	0
Mollusca	Gastropoda		Lepetodrilidae	***Lepetodrilus* sp. B**	0	1	0	1	0	0	0	0	0	0	0	0	0	0
Mollusca	Gastropoda		Lepetodrilidae	***Pseudorimula midatlantica***	0	0	0	0	0	1	1	1	1	1	1	1	0	0

Mollusca	Gastropoda		Lepetodrilidae	**_Pseduorimula_ sp.**	0	1	0	0	0	0	0	0	0	0	0	0	0	0
Mollusca	Gastropoda		Neoleptopsidae	**_Paralepetopsis ferrugivora_**	0	0	0	0	0	1	0	0	0	0	0	0	0	0
Mollusca	Gastropoda		Neoleptopsidae	**_Euleptopsis_ sp.**	0	1	0	1	0	0	0	0	0	0	0	0	0	0
Mollusca	Gastropoda		Orbitestellidae	**_Lurifax vitreus_**	0	0	0	0	0	1	0	0	0	0	0	0	0	0
Mollusca	Gastropoda		Peltospiridae	**_Chrysomallon squamiferum_**	1	1	0	1	0	0	0	0	0	0	0	0	0	0
Mollusca	Gastropoda		Peltospiridae	**_Lirapex costellata_**	0	0	0	0	0	1	0	0	0	1	0	1	0	0
Mollusca	Gastropoda		Peltospiridae	**_Lirapex_ sp.**	0	0	0	0	0	0	0	0	0	0	0	1	0	0
Mollusca	Gastropoda		Peltospiridae	**_Peltospira smaragdina_**	0	0	0	0	0	1	1	1	1	1	1	1	0	0
Mollusca	Gastropoda		Peltospiridae	**_Gigantopelta aegis_**	1	0	0	0	0	0	0	0	0	0	0	0	0	0
Mollusca	Gastropoda		Peltospiridae	**_Gigantopelta chessoia_**	0	0	0	0	0	0	0	0	0	0	0	0	1	1
Mollusca	Gastropoda		Phenacolepadidae	**_Shinkailepas briandi_**	0	0	0	0	0	1	0	0	0	0	1	1	0	0
Mollusca	Gastropoda		Phenacolepadidae	**_Shinkailepas_ sp. MAR**	0	0	0	0	0	0	0	0	0	0	0	1	0	0
Mollusca	Gastropoda		Phenacolepadidae	**_Shinkailepas_ sp. CIR**	0	1	0	1	0	0	0	0	0	0	0	0	0	0
Mollusca	Gastropoda		Provannidae	**_Alviniconcha_ sp. type 3**	0	1	1	1	0	0	0	0	0	0	0	0	0	0
Mollusca	Gastropoda		Provannidae	**_Desbruyeresia marisindica_**	0	1	0	1	0	0	0	0	0	0	0	0	0	0
Mollusca	Gastropoda		Provannidae	**Provannid sp.**	0	0	0	0	1	0	0	0	0	0	0	0	0	0
Mollusca	Gastropoda		Provannidae	**_Provanna_ n. sp. "cooki"**	0	0	0	0	0	0	0	0	0	0	0	0	1	1
Mollusca	Gastropoda		Raphitomidae	**_Phymorhynchus_ sp. CIR**	0	1	1	1	0	0	0	0	0	0	0	0	0	0
Mollusca	Gastropoda		Raphitomidae	**_Phymorhynchus_ sp. SWIR**	1	0	0	0	0	0	0	0	0	0	0	0	0	0
Mollusca	Gastropoda		Raphitomidae	**_Phymorhynchus carinatus_**	0	0	0	0	0	0	0	0	0	1	1	1	0	0
Mollusca	Gastropoda		Raphitomidae	**_Phymorhynchus_ aff. _carinatus_**	0	0	0	0	0	0	0	0	0	0	0	1	0	0
Mollusca	Gastropoda		Raphitomidae	**_Phymorhynchus moskalevi_**	0	0	0	0	0	0	0	0	1	1	1	1	0	0
Mollusca	Gastropoda		Raphitomidae	**_Phymorhynchus ovatus_**	0	0	0	0	0	1	1	1	1	1	1	1	0	0
Mollusca	Gastropoda		Skeneidae	**_Bruceiella_ sp.**	0	1	0	0	0	0	0	0	0	0	0	0	0	0
Mollusca	Gastropoda		Skeneidae	**_Protolira valvatoides_**	0	0	0	0	0	1	1	0	0	0	0	0	0	0
Mollusca	Gastropoda		Skeneidae	**_Protolira thorvaldssoni_**	0	0	0	0	0	1	1	0	0	1	0	1	0	0
Mollusca	Gastropoda		Skeneidae	**cf. _Protolira_ sp.**	0	0	0	0	0	0	0	0	0	0	0	0	1	1
Mollusca	Gastropoda		Sutulizonidae	**_Sutulizona pterodon_**	0	0	0	0	0	0	0	0	0	1	0	1	0	0
Mollusca	Gastropoda		Sutulizonidae	**_Sutulizona_ sp.**	0	1	0	0	0	0	0	0	0	0	0	0	0	0
Mollusca	Gastropoda		Xylodisculidae	**_Xylodiscula analoga_**	0	0	0	0	0	1	0	0	0	0	0	0	0	0
Mollusca	Gastropoda		-	**Split limpet sp. 1**	0	1	0	0	0	0	0	0	0	0	0	0	0	0
Mollusca	Gastropoda		-	**Split limpet sp. 2**	0	1	0	0	0	0	0	0	0	0	0	0	0	0
Mollusca	Bivalvia		Mytilidae	**_Bathymodiolus azoricus_**	0	0	0	0	0	1	1	1	0	0	0	0	0	0
Mollusca	Bivalvia		Mytilidae	**_Bathymodiolus marisindicus_**	1	1	0	1	0	0	0	0	0	0	0	0	0	0
Mollusca	Bivalvia		Mytilidae	**_Bathymodiolus puteoserpentis_**	0	0	0	0	0	0	0	1	1	1	1	0	0	0
Mollusca	Bivalvia		Thyasiridae	**_Thyasira_ sp.**	0	0	0	0	0	0	0	0	0	0	1	0	0	0
Mollusca	Bivalvia		Vesicomyidae	**_Abyssogena southwardae_**	0	0	0	0	0	0	0	0	0	0	1	0	0	0
Arthropoda	Pycnogonida		Ammotheidae	**_Sericosura heteroscela_**	0	0	0	0	0	1	0	0	0	0	0	1	0	0
Arthropoda	Pycnogonida		Ammotheidae	**_Sericosura bamberi_**	0	0	0	0	0	0	0	0	0	0	0	0	1	1
Arthropoda	Pycnogonida		Ammotheidae	**_Sericosura dimorpha_**	0	0	0	0	0	0	0	0	0	0	0	0	0	1
Arthropoda	Pycnogonida		Ammotheidae	**_Sericosura curva_**	0	0	0	0	0	0	0	0	0	0	0	0	0	1
Arthropoda	Theocostraca	Cirripedia	Chionelasmatidae	**_Eochionelasmus_ sp.**	0	1	0	1	0	0	0	0	0	0	0	0	0	0
Arthropoda	Theocostraca	Cirripedia	Eolepadidae	**_Neolepas_ sp. 1**	1	1	0	1	0	0	0	0	0	0	0	0	0	0
Arthropoda	Theocostraca	Cirripedia	Eolepadidae	**_Vulcanolepas scotiaensis_**	0	0	0	0	0	0	0	0	0	0	0	0	1	1
Arthropoda	Malacostraca	Amphipoda	Ischyroceridae	**_Bonnierella compar_**	0	0	0	0	0	1	0	0	0	0	0	0	0	0
Arthropoda	Malacostraca	Amphipoda	Amphilochidae	**_Gitanopsis alvina_**	0	0	0	0	0	1	0	0	0	0	0	0	0	0
Arthropoda	Malacostraca	Amphipoda	Calliopiidae	**_Bouvierella curtirama_**	0	0	0	0	0	1	0	0	0	0	0	0	0	0
Arthropoda	Malacostraca	Amphipoda	Pontogeneiidae	**_Luckia striki_**	0	0	0	0	0	1	0	0	0	0	0	0	0	0
Arthropoda	Malacostraca	Amphipoda	Phoxocephalidae	**_Harpinia pico_**	0	0	0	0	0	0	1	0	0	0	0	0	0	0
Arthropoda	Malacostraca	Amphipoda	Stegocephalidae	**_Steleuthera ecoprophycea_**	0	0	0	0	0	0	0	0	0	1	0	0	0	0
Arthropoda	Malacostraca	Decapoda	Alvinocarididae	**_Alvinocaris markensis_**	0	0	0	0	0	1	1	1	1	1	1	0	0	0
Arthropoda	Malacostraca	Decapoda	Alvinocarididae	**_Shinkaicaris_ sp.**	0	1	0	0	0	0	0	0	0	0	0	0	0	0
Arthropoda	Malacostraca	Decapoda	Alvinocarididae	**_Chorocaris chacei_**	0	0	0	0	0	1	1	0	1	1	1	1	0	0
Arthropoda	Malacostraca	Decapoda	Alvinocarididae	**_Chorocaris_ sp.**	1	1	0	1	0	0	0	0	0	0	0	0	0	0
Arthropoda	Malacostraca	Decapoda	Alvinocarididae	**_Mirocaris indica_**	1	1	1	1	0	0	0	0	0	0	0	0	0	0
Arthropoda	Malacostraca	Decapoda	Alvinocarididae	**_Mirocaris fortunata_**	0	0	0	0	0	1	1	1	1	1	1	1	0	0
Arthropoda	Malacostraca	Decapoda	Alvinocarididae	**_Rimicaris kairei_**	1	1	1	1	1	0	0	0	0	0	0	0	0	0
Arthropoda	Malacostraca	Decapoda	Alvinocarididae	**_Rimicaris exoculata_**	0	0	0	0	0	1	1	1	1	1	1	1	0	0
Arthropoda	Malacostraca	Decapoda	Bythograeidae	**_Austinograea rodriguez_**	0	1	1	1	1	0	0	0	0	0	0	0	0	0
Arthropoda	Malacostraca	Decapoda	Bythograeidae	**_Segonzacia mesatlantica_**	0	0	0	0	0	1	1	1	1	1	1	1	0	0
Arthropoda	Malacostraca	Decapoda	Munidopsidae	**_Mundiopsis_ sp. SWIR**	1	0	0	0	0	0	0	0	0	0	0	0	0	0
Arthropoda	Malacostraca	Decapoda	Munidopsidae	**_Munidopsis laticorpus_**	0	1	0	1	0	0	0	0	0	0	0	0	0	0
Arthropoda	Malacostraca	Decapoda	Munidopsidae	**_Munidopsis exuta_**	0	0	0	0	0	1	1	1	1	1	1	1	0	0
Arthropoda	Malacostraca	Decapoda	Kiwaidae	**_Kiwa tyleri_**	0	0	0	0	0	0	0	0	0	0	0	0	1	1
Arthropoda	Malacostraca	Decapoda	Kiwaidae	**_Kiwa_ n. sp. "SWIR"**	1	0	0	0	0	0	0	0	0	0	0	0	0	0
Arthropoda	Malacostraca	Decapoda	Nephropidae	**_Thymopides laurentae_**	0	0	0	0	0	0	0	0	0	1	0	0	0	0
Arthropoda	Malacostraca	Isopoda	Ischnomesidae	**_Heteromesus calcar_**	0	0	0	0	0	1	0	0	0	0	0	0	0	0
Arthropoda	Malacostraca	Isopoda	Ischnomesidae	**_Heteromesus ctenobasius_**	0	0	0	0	0	1	0	0	0	0	0	0	0	0
Arthropoda	Malacostraca	Tanaidacea	Paratanoidea incertae sedis	**_Armaturatanais atlanticus_**	0	0	0	0	0	1	0	0	0	0	0	0	0	0

Arthropoda	Malacostraca	Tanaidacea	Colletteidae	***Leptognathiella fragilis***	0	0	0	0	0	1	0	0	0	0	0	0	0	0
Arthropoda	Malacostraca	Tanaidacea	Leptocheliidae	***Mesotanais styxis***	0	0	0	0	0	1	0	0	0	0	0	0	0	0
Arthropoda	Malacostraca	Tanaidacea	Nototanaidae	***Obesutanais sigridi***	0	0	0	0	0	1	0	0	0	0	0	0	0	0
Arthropoda	Malacostraca	Tanaidacea	Typhlotanaidae	***Typhlotanais incognitus***	0	0	0	0	0	1	0	0	0	0	0	0	0	0
Echinodermata	Asteroidea		Paulasteridae	***Paulasterias tyleri***	0	0	0	0	0	0	0	0	0	0	0	0	1	1
Echinodermata	Ophiuroidea		Ophiuridae	***Ophioctenella acies***	0	0	0	0	0	1	1	1	1	1	1	1	0	0
Echinodermata	Holothuroidea		Chiridotidae	***Chiridota* sp.**	1	0	0	0	0	0	0	0	0	0	0	0	0	0
Echinodermata	Holothuroidea		-	**Apodacean sp.**	0	0	0	1	0	0	0	0	0	0	0	0	0	0
Chaetognatha	Sagittoidea		Spadellidae	***Calispadella alata***	0	0	0	0	0	1	0	0	0	0	0	0	0	0
Chordata	Actinopteri		Synaphobranchidae	***Ilyophis saldanhai***	0	0	0	0	0	0	0	1	0	0	0	0	0	0
Chordata	Actinopteri		Lotidae	***Gaidropsaurus* sp.**	0	0	0	0	0	1	0	0	0	0	0	0	0	0
Chordata	Actinopteri		Zoarcidae	***Pachycara thermophilum***	0	0	0	0	0	0	0	1	1	1	0	1	0	0
Chordata	Actinopteri		Zoarcidae	***Pachycara saldanhai***	0	0	0	0	0	1	1	0	0	0	0	0	0	0
				Total species at each vent field:	21	35	9	21	4	47	23	18	17	25	20	32	12	14
